# Adaptability to High Temperature and Stay-Green Genotypes Associated With Variations in Antioxidant, Chlorophyll Metabolism, and γ-Aminobutyric Acid Accumulation in Creeping Bentgrass Species

**DOI:** 10.3389/fpls.2021.750728

**Published:** 2021-10-28

**Authors:** Zhou Li, Mingyan Tang, Muhammad Jawad Hassan, Yan Zhang, Liebao Han, Yan Peng

**Affiliations:** ^1^College of Grassland Science and Technology, Sichuan Agricultural University, Chengdu, China; ^2^Institute of Turfgrass Science, Beijing Forestry University, Beijing, China

**Keywords:** resource evaluation, thermotolerance, oxidative damage, senescence (leaf), metabolism, environmental stress

## Abstract

High temperature limits the cultivation and utilization of cool-season plants in many regions worldwide. Recently, extreme hot waves swept across the globe in summer, leading to enormous economic loss. The evaluation and identification of genotypic variation in thermotolerance within species are critical to breeding for environmental adaptation and also provide potential materials to explore thermo-resistant mechanism in plants. Forty-two accessions of creeping bentgrass (*Agrostis stolonifera*), which is a cool-season perennial grass for turf and ecological remediation, were collected from 15 different countries. Physiological traits, namely, chlorophyll (Chl) content, electrolyte leakage, photochemical efficiency, performance index on absorption basis, leaf relative water content, and osmotic potential were used to evaluate the heat tolerance of these materials in controlled growth chambers and field during summer. Stay-green and early-aging genotypes were selected to further reveal the potential mechanism of tolerance to senescence and heat damage associated with alterations in Chl metabolism, antioxidant and photosynthetic capacity, and endogenous γ-aminobutyric acid (GABA). Findings showed that there were significant genetic variations in physiological traits among 41 materials in response to high temperature stress. The 13M, PROVIDENCE, and LOFTS L-93 were the top three accessions with superior tolerance to heat and summer stress than other materials in terms of laboratory and field tests. In response to heat stress, the stay-green genotype PROVIDENCE exhibited significantly higher photochemical efficiency, net photosynthetic rate, transpiration rate, and water use efficiency than the heat-susceptible W6 6570. Delayed leaf senescence in relation to less Chl loss was detected in the PROVIDENCE associated with maintenance of significantly higher expression levels of Chl-anabolic genes (*AsCHLH, AsPBGD*, and *AsPOR*) and lower Chl-catabolic gene *AsPPH* under heat stress. Genetic attributes, such as better capacity to scavenge reactive oxygen species and higher endogenous GABA content could play positive roles in alleviating heat-induced senescence, oxidative damage, and metabolic disturbance in the PROVIDENCE.

## Introduction

Plants often suffer from various environmental stresses in nature; even cultivated crops under intensive care are easily affected by adverse environmental conditions. Extreme high temperature occurs frequently across the whole world and poses a major challenge to the cultivation and utilization of most crops in temperate regions (Sun et al., 2019). Because of the predicted rise in global temperature, heat stress-induced loss in crop economy will further expand worldwide (Van Vuuren et al., 2009). The identification of genetic variation in thermotolerance in one particular plant species is an essential requirement for the breeding and cultivation of a new variety. Previous studies have shown that wheat (*Triticum aestivum*) genotypes exhibited a significant variation in heat tolerance, and that these diverse genotypes could be used in wheat breeding programs for heat tolerance (Ahamed et al., 2010; Thistlethwaite et al., 2015; Sharma et al., 2016). As the third most abundant flowering plants, grass species exhibit extensive adaptation to abiotic stress and variations in stress tolerance, such as thermotolerance (Huang et al., 2014). The study of Sun et al. (2014) demonstrated that a higher genotypic variation in heat tolerance was found in gramineous tall fescue (*Festuca arundinacea*) species through evaluating 120 accessions from America, European, Africa, and Asia. Creeping bentgrass (*Agrostis stolonifera*), a perennial herb with genetic diversity, is widely distributed worldwide and used for lawn and forage. However, creeping bentgrass is a cool-season grass mainly adapting to cool-humid climate (Huang and Gao, 2000). The evaluation and identification of genotypic variation in thermotolerance within the species is critical to the breeding for stress tolerance and also provide potential materials to explore thermo-resistant mechanism in perennial plant species.

The stay-green genotype is regarded as an important trait for horticultural crops such as turfgrass. Heat stress accelerates leaf senescence associated with chlorophyll (Chl) degradation and loss (Jespersen et al., 2016). It has been reported that alterations in gene expression or enzyme activities responsible for Chl synthesis or degradation affected heat-induced senescence in different plants species. For example, the downregulation of Chl catabolic genes, such as *LpPPH* encoding pheophytin pheophorbide hydrolyase by exogenous chemicals calcium dichloride, salicylic acid, and urea significantly alleviated heat-induced leaf senescence of perennial ryegrass (*Lolium perenne*) (Jing et al., 2019). Persistent heat stress accelerated leaf chlorosis of white clover (*Trifolium repens*) positively related to increases in transcript levels of *chlorophyllase* (*CHLASE*) and *pheophorbide a oxygenase* (*PAO*) genes for Chl degradation (Luo et al., 2020). The inhibition of Chl biosynthesis, as reflected by significant declines in activities of Chl-anabolic enzymes such as porphobilinogen deaminase (PBGD), magnesium-chelatase (Mg-CHT), and protochlorophyllide reductase (POR), was correlated with a significant increase in heat-induced leaf senescence of cucumber (*Cucumis sativus*) (Tewari, 1998). In bentgrass species, thermal rough bentgrass (*Agrostis scabra*) exhibited a significant delay in leaf senescence associated with lower Chl-degrading enzyme chlorophyllase (CHLASE) activity when compared with creeping bentgrass, but there was no significant difference in PBGD activity after 42 days of heat stress (Rossi et al., 2017). The maintenance of higher Chl content in leaves is propitious to growth and mitigation of photoinhibition for most of crops during hot summer, since Chl is the most important pigment for light absorption and conversion (Chen, 2014). Chl degradation induced by heat stress slows down photosynthetic process, leading to growth retardation and yield loss (Lipiec et al., 2013). Higher Chl content was found to be the key indicator of better heat and drought tolerance in wheat genotypes (Rehman et al., 2016). In addition, the overaccumulation of reactive oxygen species (ROS) causes oxidative damage to chloroplast, mitochondria, and other organelles, contributing to early aging and cell death under adverse environmental conditions, such as a long period of high temperature in nature (Parent et al., 2008). The study of Jing et al. (2019) demonstrated that excessive ROS accumulation resulted in oxidative damage to Chl under heat stress, which aggravated leaf senescence.

Transitional and warm climatic regions limit the intensive cultivation of cool-season plants and increase the maintenance and management cost of many horticultural crops during summer (Malhotra, 2017). Some earlier studies have evaluated differences in heat tolerance of creeping bentgrass, but were restricted to less cultivars or materials. In this study, 42 creeping bentgrass accessions were collected from 15 different countries. Physiological traits, namely, Chl content, electrolyte leakage (EL), photochemical efficiency (Fv/Fm), performance index on absorption basis (PI_ABS_), leaf relative water content (RWC), and osmotic potential (OP) were used for evaluating the heat tolerance of these materials in controlled greenhouse and field during summer. Stay-green and early-aging genotypes were selected to further reveal the potential mechanism of heat tolerance associated with alterations in Chl metabolism, antioxidant and photosynthetic capacity, and content of endogenous γ-aminobutyric acid (GABA), a crucial metabolite involved in Chl metabolism and heat tolerance in plants (Li et al., 2016, 2021). Comparing and identifying these genotypes differing in heat tolerance under field conditions during summer and in controlled greenhouses will provide more accurate information for the breeding and utilization of creeping bentgrass species.

## Materials and Methods

### Plant Materials and Treatments

Forty-two creeping bentgrass materials were collected from National Plant Germplasm System (NPGS) in the United States and Beijing Zhengdao Ecology Science and Technology Ltd. These materials are distributed in or have originated from 15 different countries (Supplementary Table 1). In March 2019, 30 seeds of each material were sown in a cylindrical plastic container (11 cm in diameter and 33 cm in length) containing sand and soil (1:1, w:w). All the materials were placed in a greenhouse [average 24/17°C (day/night), 65% relative humidity, and 700 μmol·m^−2^·s^−1^ photosynthetically active radiation (PAR)]. After 7 days of germination, seedlings were watered using half-strength Hoagland's solution (Hoagland and Arnon, 1950) once a week and trimmed twice or thrice a week to maintain a canopy height of about 3 cm. All the materials were cultivated in the greenhouse for 2 months until grasses had covered the whole mouth of the pipe. Grasses were moved into a walking chamber for 7 days of acclimation [23/18°C (day/night), 65% relative humidity, and 700 μmol·m^−2^·s^−1^PAR]. The plants were then divided into two groups: one group was placed in a normal walking chamber (the same as above), and the other was placed in a walking chamber with high temperature [37/30°C (day/night), 65% relative humidity, and 700 μmol·m^−2^·s^−1^ PAR]. All the materials were completely arranged in walking chambers, and each material included four biological replicates (four containers). Leaf samples were taken after 30 days of normal cultivation and heat stress.

For the evaluation of heat tolerance of 18 materials in the field, the sod of each material (5 cm in diameter) was planted in the Research Farm of Sichuan Agricultural University (Chongzhou, Sichuan, China, east longitude 103°07′-103°49′ and north latitude 30°30′-30°53′) in September 2019. All the materials were watered by half-strength Hoagland's solution once a week and trimmed twice a week to maintain a canopy height of about 3–5 cm until each material formed a circular turf with 40 cm in diameter. For physiochemical properties, soil pH, total nitrogen, available phosphate, available potassium, and organic matter was 6.3, 2.03 g/kg, 10.2 mg/kg, 101.1 mg/kg, and 37.6 g/kg, respectively. All the materials were completely arranged in the field, and each included four biological replicates. The identification of heat tolerance was conducted in the summer of 2020. From June 1 to July 31, the total number of daily maximum temperature over 30°C amounted to 32 days, and the maximum temperature was 37.3°C on July 26 (Supplementary Figure 1). Volumetric soil water content was maintained at a 70–80% field capacity to avoid drought. The leaf samples were collected on June 1, June 28, and July 31, 2020. The 18 materials with better heat tolerance were selected based on the first experiment that was conducted in the walking chambers.

For the comparative analysis of heat tolerance between heat-sensitive (W6 6570) and heat-tolerant (PROVIDENCE) materials, the sod of each material (5 cm in diameter) was planted in a cylindrical plastic container (11 cm in diameter and 33 cm in length) for 2 months in the greenhouse (cultivation condition and maintenance measure were the same as those mentioned above). After being acclimated in growth chambers for 7 days, the grasses were divided into two groups: one group was placed in a normal growth chamber [23/18°C (day/night), 65% relative humidity, and 700 μmol·m^−2^·s^−1^ PAR], and the other group was placed in a growth chamber with high temperature [38/33°C (day/night), 65% relative humidity, and 700 μmol·m^−2^·s^−1^ PAR] for 15 days. All the materials were completely arranged in the growth chambers, and each included four biological replicates (four containers). The leaf samples were taken at 0 and 3 h, and 3, 10, and 15 days of normal cultivation and heat stress. The selection of the two materials was based on the comprehensive evaluation of the first experiment in the walking chambers and the second experiment in the field.

### Measurements of Turf Quality, Water Status, and Photosynthetic Parameters

The evaluation of turf quality (TQ) was based on color, density, and uniformity of turf with nine scores for fully turgid and green turf, six scores for minimal acceptable level, and one score for completely dry and yellow turf (Beard and Batten, 2001). For leaf RWC, fresh leaves were mown from plants and weighed to get the fresh weight (FW), and these leaves were then soaked in deionized water for 12 h. After being taken out and blotted dry, the turgid weight (TW) of the leaves was recorded. The leaves were dried at 105°C for 20 min and later at 80°C until the dry weight (DW) became constant. The RWC was calculated based on the formula RWC (%) = [(FW–DW)/(TW–DW)] × 100% (Barrs and Weatherley, 1962). For the determination of OP, the fresh leaves were hydrated in deionized water for 12 h and the fully turgid leaves were frozen in liquid nitrogen for 10 min. When the frozen leaves were thawed in ice bath, the sap in the leaves was pressed and then inserted (10 μl) into an osmometer (Wescor, Inc., Logan, UT, United States) to get osmolality (mmol kg^−1^) and the OP = –([osmolality] × [0.001] × [2.58]) (Blum, 1989). Chl content was determined according to the method of Amnon (1949). Fv/Fm and PI_ABS_ were detected by a Chl fluorescence system (Pocket PEA) before the leaves were clipped with leaf clips for 20-min dark adaptation. A portable photosynthesis system (CIRAS-3; PP Systems, United States) was used for detecting net photosynthesis rate (Pn), transpiration rate (Tr), and water use efficiency (WUE). The leaf chamber (400 μl L^−1^ CO_2_ and 800 μmol photon m^−2^ red and blue light) was overspread with a single layer of leaves, and readings were recorded after the readings became stable.

### Measurements of Oxidative Damage, Antioxidant Capacity, and Cell Membrane Stability

For the determination of superoxide anion (${\text{O}}_{2}^{\cdot -}$) radical content, 0.1 g of leaves were homogenated with 1.5 ml of 65 mM phosphate buffered saline (PBS, pH 7.8), and the mixture was centrifuged at 10,000 g for 30 min at 4°C. The supernatant, 0.5 ml, was mixed with 0.5 ml of PBS and.1 ml of 10 mM hydrochloride, and then incubated at 25°C for 20 min. Sulfanilamide 0.5 ml and 58 mM and 0.5 ml of 7 mM a-naphthylamine were added into the mixture and then incubated at 25°C for 20 min. Chloroform 2 ml was added and shaken up. The absorbance of the chloroform was measured at 530 nm (Elstner and Heupel, 1976). The hydrogen peroxide (H_2_O_2_) content was detected using the method of Velikova et al. (2000). For the evaluation of malondialdehyde (MDA) content, the method of Dhindsa et al. (1981) was used. An assay kit (ABTS-1-D; Suzhou Comin Biotechnology Co., Ltd., China) was used for determining total antioxidant capacity (TAC) according to the instructions of the manufacturer, and 0.1 g of fresh leaves were used. For the determination of EL, 0.1 g of the fresh leaves were immersed in 35 ml deionized water at 24°C for 24 h, and the conductance of leaching liquor was detected using a conductivity meter (YSI Model 32; Yellow Springs Instrument Co., United States) as the initial conductivity (C_1_). The leaves and a solution were heated at 100°C for 30 min, and the conductance of the solution was detected as the final conductivity (C_2_). EL was then calculated based on the formula EL (%) = C_1_/C_2_ × 100% (Blum and Ebercon, 1981).

### Measurements of Endogenous GABA Content

Endogenous GABA content was detected by enzyme-linked immunosorbent assay (ELISA), and the assay kit (ml-E3042) was purchased from Shanghai Enzyme-linked Biotechnology Co. (Shanghai, China). Briefly, 0.1 g of the fresh leaves were ground with 2 ml of PBS (pH 7.4), and the mixture was centrifuged at 3,000 g for 20 min. The supernatant was used for the determination of the GABA content according to the instructions of the manufacturer.

### Gene Expression Analysis

Gene expression levels were determined by real-time quantitative polymerase chain reaction (qRT-PCR). The total RNA in the leaves was extracted using RNeasy Mini Kit (Qiagen, Germany) and according to the instructions of the manufacturer. These RNAs were reverse-transcribed to cDNA using the Revert Aid First Stand cDNA Synthesis Kit (Fermentas, Lithuania). For the determination of gene expression levels, an iCycleriQ (Bio-Rad, United States) qRT-PCR detection system with SYBR Green Supermix (Bio-Rad) was used. The conditions of the PCR protocol for all the genes, namely, *As*β*-actin* (internal reference gene), Chl-metabolism-related genes (*AsPBGD, AsCHLH-*encoding magnesium-chelatase H subunit, *AsPPH*, and *AsPOR*), *AsGAD1-*encoding glutamate decarboxylase involved in GABA biosynthesis, and senescence-associated genes (*As120, Ash12, AsSAG12*, and *AsSAG39*) were as follows: 5 min at 95 C and 40 repeats of denaturation at 95°C for 15 s, annealing at 55–60°C (Supplementary Table 2) for 45 s, followed by heating the amplicon from 60 to 95°C to obtain the melting curve. Primer sequences of all the genes are shown in Supplementary Table 2. Transcript levels of all the genes were calculated based on the formula 2^−ΔΔCt^ described by Kenneth and Thomas (2002).

### Statistical Analysis

Significant differences among treatments were tested by Fisher's protected least significance (LSD) test at a 0.05 probability level, and Pearson correlation analysis was performed at a 0.05 and 0.01 probability level (SAS 9.1; SAS Institute, Cary, NC, United States). Subordinate function values analysis (SFVA) was performed for a comprehensive evaluation of heat tolerance based on different physiological parameters. If a parameter had a positive relationship with heat tolerance, the formula U_ij_ = (X_ij_-X_jmin_)/(X_jmax_-X_jmin_) was used. If a parameter had a negative relationship with heat tolerance, the formula U_ij_ = 1–(X_ij_-X_jmin_)/(X_jmax_-X_jmin_) was used. X_ij_ indicated the difference of mean value of the parameter j between normal condition and heat stress in material i. X_jmin_ indicated the mimimum difference of mean value of the parameter j between normal condition and heat stress in material i. X_jmax_ indicated the maximum difference of mean value of the parameter j between normal conditions and heat stress in material i. The comprehensive evaluation index of heat tolerance was calculated based on the formula B = ∑U_ij_/n. U_ij_ indicated the comprehensive score of the parameter j in material i. n indicated the total number of physiological parameters used for evaluating heat tolerance. The larger SFVA value, the better the heat tolerance. Hierarchical clustering (heat map) of physiological parameters was performed using the R statistical software (R3.5.2 by R Development Core Team).

## Results

### Evaluation of Heat Tolerance Among the 41 Materials in the Controlled Greenhouse

There was an obvious difference among 41 materials in leaf RWC, OP, EL, Chl content, and PI_ABS_ between control and heat stress (Supplementary Figures 1, 2). The range of variation in difference in leaf RWC was from 4.2 to 44.3% (Supplementary Figure 1A). Maximum difference in RWC was observed in W6 6570 (44.3%), followed by SEASIDE 2 (28.9%) and W6 6569 (27.1%). KLONOBAJA (4.2%) and SMALL LIZARD (4.2%) exhibited a minimum variation in RWC (Supplementary Figure 1A). For the change in OP, the variation ranged from 0.01 to 0.72 MPa (Supplementary Figure 1B). The variation in OP of three materials (O93, W6 6574, and F-251) was less than 0.1 MPa; however, SR 1020 showed highest difference in OP (0.72 MPa) (Supplementary Figure 1B). A 1.11–62.41% variation range was detected in EL among 41 materials. Minimum variation was found in PENN WAY (1.1%) and the second to last was PA1 (1.9%), whereas top three variations were found in the PENNEAGLE (46.4%), PENNCROSS (46.0%), and EMERALD (45.2%), respectively (Supplementary Figure 2A). Heat stress caused a significant decline in Chl content of all the materials, and decreasing range was between 0.70 and 8.01 mg g^−1^ (Supplementary Figure 2B). W6 6569 showed a maximum decline in Chl content, whereas TALEH (0.7 mg g^−1^) showed a minimum decrease in Chl content, followed by W6 6569 (1.1 mg g^−1^) and LOFTS L-93 (1.7 mg g^−1^) (Supplementary Figure 2B). The most significant decline in PI_ABS_ was observed in PENNEAGLE, and minimum values were found in NATIONAL and MSCB-11 (Supplementary Figure 2C).

**Figure 1:**
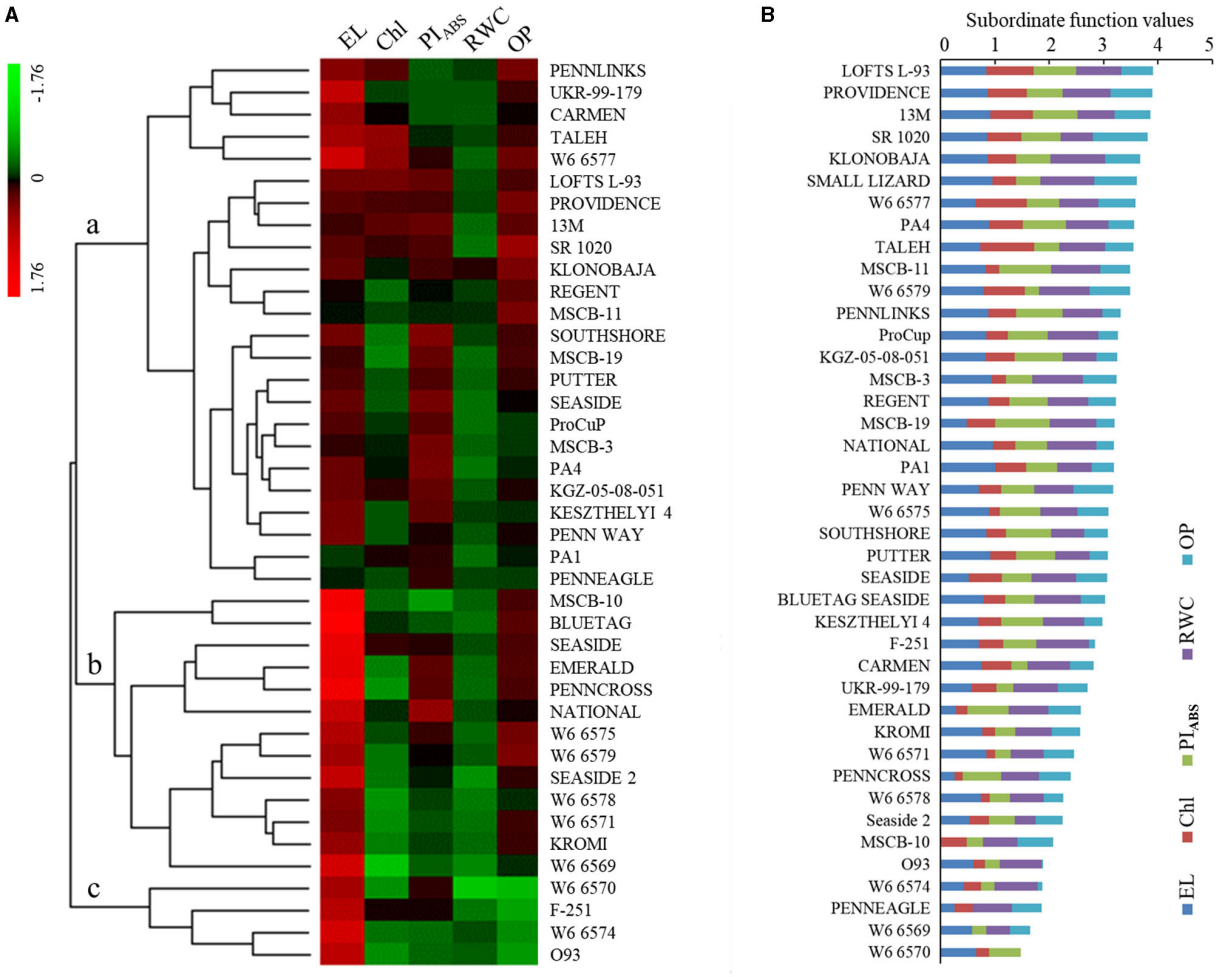
Changes in **(A)** heat map that was made by log_2_FC (fold change of heat stress in relation to control under normal conditions) and **(B)** the subordinate function values analysis of five different physiological parameters for comprehensive evaluation of heat tolerance in 41 creeping bentgrass materials under heat stress in the controlled greenhouse.

Hierarchical clustering of five physiological parameters (EL, Chl, PI_ABS_, RWC, and OP) among the 41 materials were mainly divided into three subclusters (a, b, and c) in response to heat stress (Figure 1A). The parameters in the subcluster showed less changes under heat stress. EL, Chl, and RWC exhibited greater changes, but PI_ABS_ and OP showed less changes in the subcluster b. Most parameters in the subcluster c exhibited greater changes under heat stress (Figure 1A). Based on the SFVA, the heat tolerance of the 41 materials was ranked, and higher SFV means better heat tolerance (Figure 1B). LOFTS L-93 (top), PROVIDENCE (second), and 13M (third) had better heat tolerance than the other materials. PENNEAGLE, W6 6569, and W6 6570 exhibited less heat tolerance than the other 38 materials (Figure 1B). The Pearson correlation analysis found that the subordinate function value (SFV) was significantly correlated with EL, Chl, PI_ABS_, RWC, or OP under heat stress (Table 1). The SFV and Chl had the highest correlation coefficient (*r* = 0.681). EL, PI_ABS_, RWC, and OP also showed high correlation coefficients with the SFV (Table 1).

**Table 1:** Pearson correlation coefficient analysis based on six parameters, namely, mean subordinate function value (SFV), electrolyte leakage (EL), chlorophyll (Chl) content, performance index on absorption basis (PI_ABS_), leaf relative water content (RWC), and osmotic potential (OP) among 41 creeping bentgrass accessions under heat stress.

	**SFV**	**EL**	**Chl**	**PI_**ABS**_**	**RWC**	**OP**
SFV	1					
EL	−0.575^“**”^	1				
Chl	0.681^“**”^	−0.151	1			
PIABS	0.565^“**”^	−0.366^“*”^	0.185	1		
RWC	0.565^“**”^	−0.175	0.335^“*”^	0.046	1	
OP	0.583^“**”^	−0.028	0.326^“*”^	0.089	0.429^“**”^	1

“*” or “**” *indicates significance at P < 0.05 or P < 0.01, respectively*.

### Evaluation of Heat Tolerance Among 18 Materials in the Field in Summer

**Figure 2:**
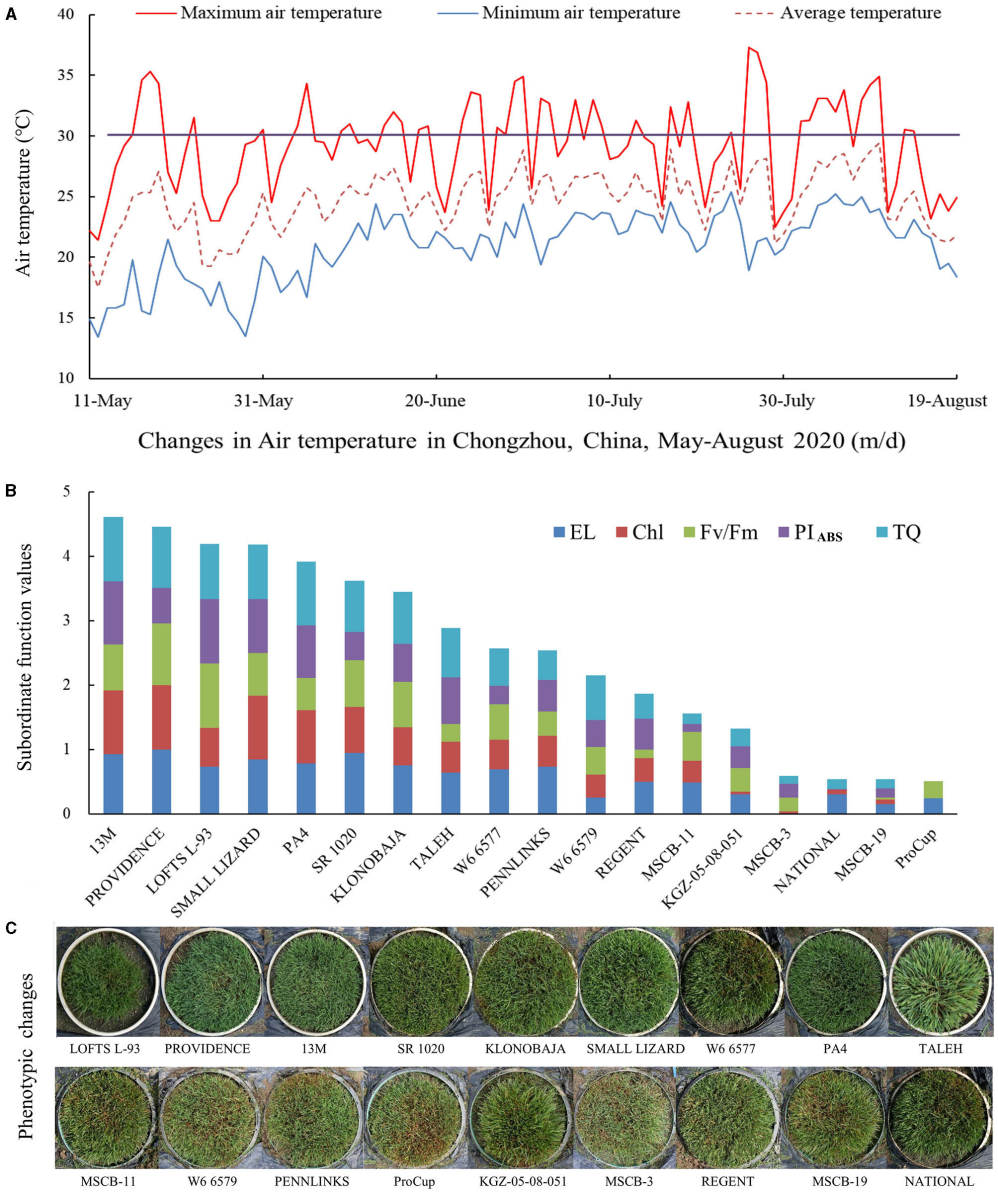
Changes in **(A)** daily maximum, minimum, and average air temperature, **(B)** the subordinate function values analysis of five different physiological parameters for comprehensive evaluation of heat tolerance, and **(C)** phenotypes of 18 creeping bentgrass materials during summer in 2020 under field conditions.

During summer, from June 1 to July 31, 2020, daily maximum average temperature ranged from 22.5 to 37.3°C, and daily maximum average temperature above 30°C amounted to 32 days under field conditions (Figure 2A). In addition, there was a total of 11 days of daily maximum average temperature between 28 and 30°C (Figure 2A). High temperature stress in summer induced gradual decline in TQ of 18 materials with better tolerance selected from 41 materials based on the experiment conducted in the controlled greenhouse (Supplementary Figure 3A). On July 31, PROVIDENCE, 13M, PA4, and SMALL LIZARD showed higher TQ, but MSCB-19, KGZ-05-08-051, ProCup, and MSCB-3 showed lower TQ (Supplementary Figure 3A). On the contrary, a significant increase in EL was observed in all the materials (Figure 2C). SMALL LIZARD, PA4, 13M, PROVIDENCE exhibited lower EL than the other materials on June 28 and July 31 (Supplementary Figure 3B). Chl content, Fv/Fm, and PI_ABS_ in the leaves decreased quickly during summer time (Supplementary Figures 4A–C). KLONOBAJA, TALEH, 13M, and SMALL LIZARD maintained higher Chl as compared with the remaining 14 materials (Supplementary Figure 4A). Decline in Fv/Fm was least in PROVEIDENCE and second lowest in LOFTS L-93 after summer (Supplementary Figure 4B). LOFTS L-93, KLONOBAJA, and 13M exhibited significantly higher PI_ABS_ than the other materials in summer (Supplementary Figure 4C). Based on the SFVA of four parameters (TQ, EL, CHL, Fv/Fm, and PI_ABS_), the heat tolerance of the 18 materials was ranked (Figure 2B). 13M exhibited strongest heat tolerance, followed by PROVIDENCE (second) and LOFTS L-93 (third). The heat tolerance of ProCup was worst. NATIONAL and MSCB-19 had the same score, and their heat tolerance was next to last among the 18 materials under field conditions in summer (Figure 2B). Figure 2C shows the phenotypic changes in the 18 materials during summer under field conditions. Based on comprehensive evaluations in the controlled greenhouse and under field conditions, 13M, PROVIDENCE, and LOFTS L-93 were the top three materials with heat tolerance better than that of the other materials (Figures 1B, 2B).

### Differential Responses to Heat Stress in Photosynthesis and Chlorophyll Metabolism Between Heat-Tolerant and Heat-Sensitive Materials in Controlled Growth Chambers

**Figure 3:**
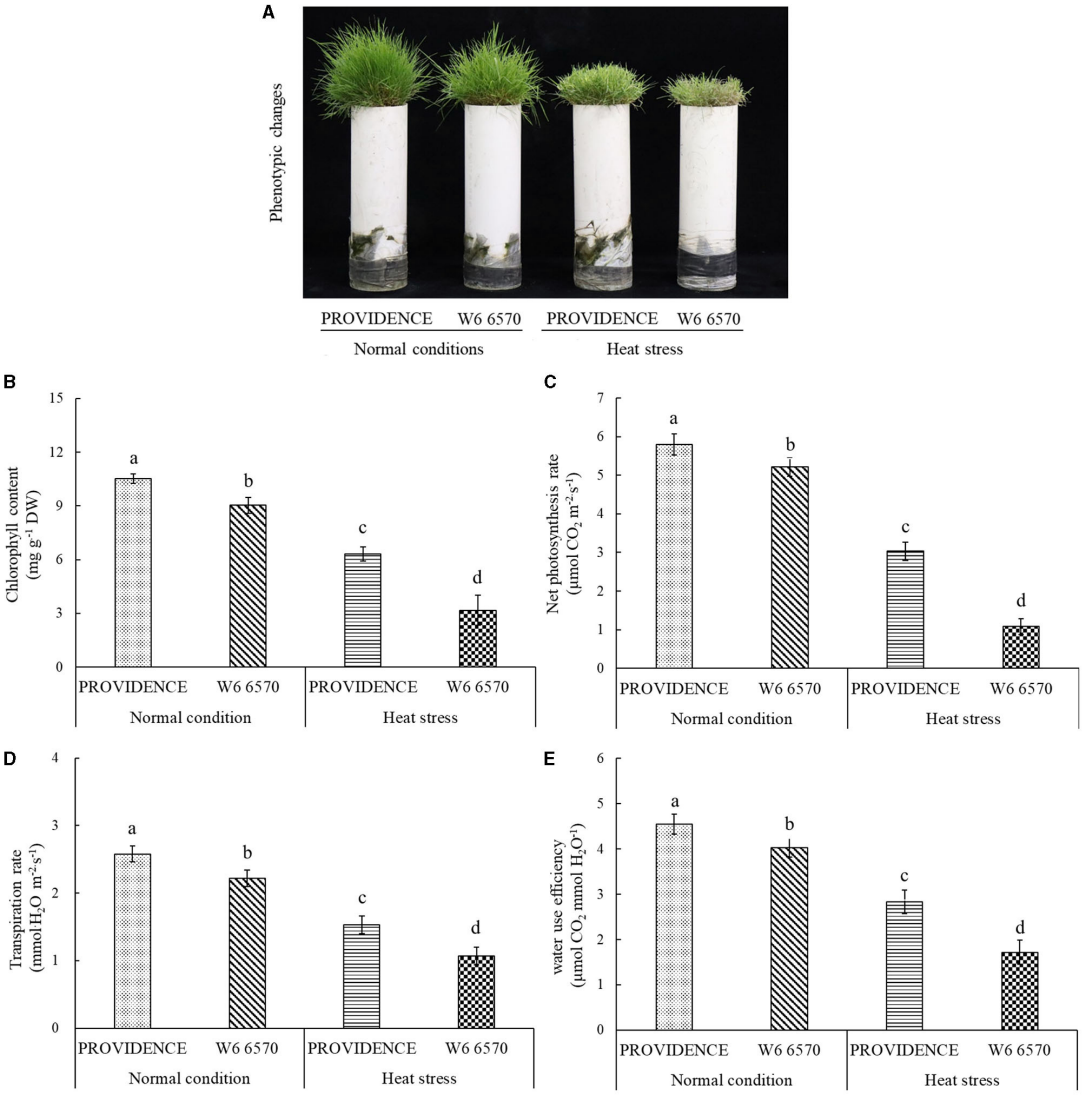
Changes in **(A)** phenotype, **(B)** chlorophyll content, **(C)** net photosynthetic rate, **(D)** transpiration rate, and **(E)** water use efficiency in leaves of two creeping bentgrass materials (PROVIDENCE and W6 6570) in response to 15 days of heat stress (38/33°C, day/night) in controlled growth chambers. Vertical bars indicate ± standard error (SE) of mean (*n* = 4), and different letters above column indicate significant differences (*P* ≤ 0.05).

**Figure 4:**
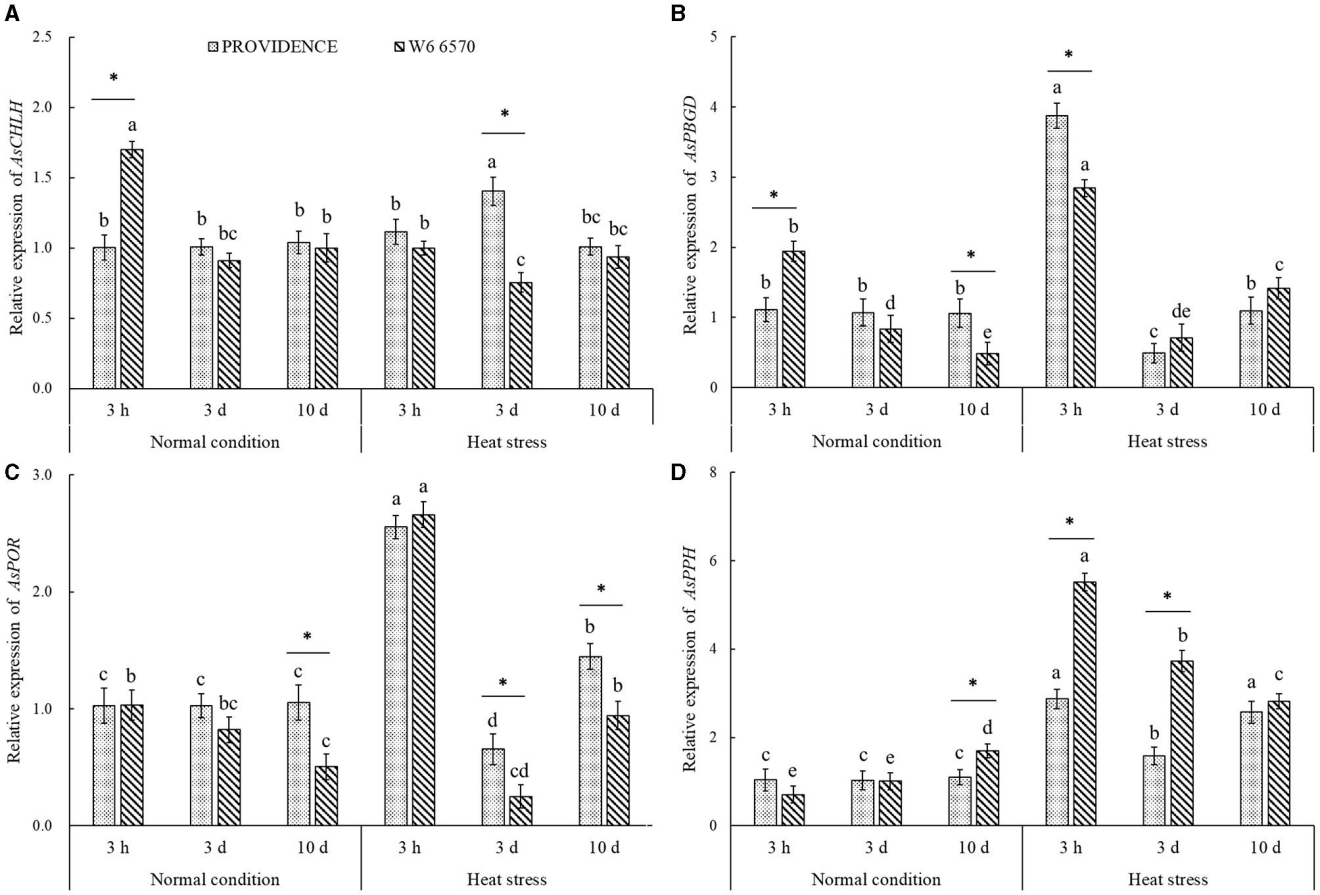
Changes in transcript levels of **(A)** *AsCHLH*, **(B)** *AsPBGD*, **(C)** *AsPOR*, and **(D)** *AsPPH* in leaves of two creeping bentgrass materials (PROVIDENCE and W6 6570) during heat stress (38/33°C, day/night) in controlled growth chambers. Vertical bars indicate ± SE of mean (*n* = 4). Different letters above column indicate significant differences (*P* ≤ 0.05) in a particular material (PROVIDENCE or W6 6570) under normal conditions and heat stress. The asterisk “*” indicates significant differences (*P* ≤ 0.05) between PROVIDENCE and W6 6570 at one specific time point (3 h, 3 days, or 10 days).

For the comparison experiment in controlled growth chambers, heat stress (15 days) made the leaves of heat-tolerant PROVIDENCE and heat-sensitive W6 6570 turn into yellow and also hindered their growth; however, heat stress had minor adverse effects on PROVIDENCE (Figure 3A). Chl content, Pn, Tr, and WUE in both PROVIDENCE and W6 6570 declined significantly under heat stress (Figures 3B–E). Obviously, PROVIDENCE exhibited higher Chl content, Pn, Tr, and WUE than W6 6570 under normal conditions and heat stress (Figures 3B–E). For the expression level of genes involved in Chl metabolism, the expression of *AsCHLH* was significantly higher in W6 6570 than in PROVIDENCE at 3 h, but was not different at 3 and 10 d under normal conditions (Figure 4A). Heat stress significantly decreased the expression of *AsCHLH* in W6 6570, but increased its expression in PROVIDENCE at 3 days (Figure 4A). Under normal conditions, the expression of *AsPBGD* gradually declined from 3 h to 10 d in W6 6570, but did not change in PROVIDENCE (Figure 4B). Under heat stress, PROVIDENCE exhibited a significantly higher *AsPBGD* expression than W6 6570 at 3 h (Figure 4B). The expression of *AsPOR* did not vary in PROVIDENCE, but gradually declined in W6 6570 under normal conditions (Figure 4C). PROVIDENCE had a significantly higher *AsPOR* expression than W6 6570 at 10 days under normal conditions and at 3 and 10 days under heat stress (Figure 4C). Heat stress significantly upregulated the expression of *AsPPH* in the leaves of PROVIDENCE and W6 6570; however, the expression of *AsPPH* was significantly lower in PROVIDENCE as compared with that in W6 6570 at 3 h and 3 days of heat stress (Figure 4D).

### Differential Responses to Heat Stress in Antioxidant Capacity, γ-Aminobutyric Acid, and Senescence-Associated Genes Expression Between Heat-Tolerant and Heat-Sensitive Materials in Controlled Growth Chambers

**Figure 5:**
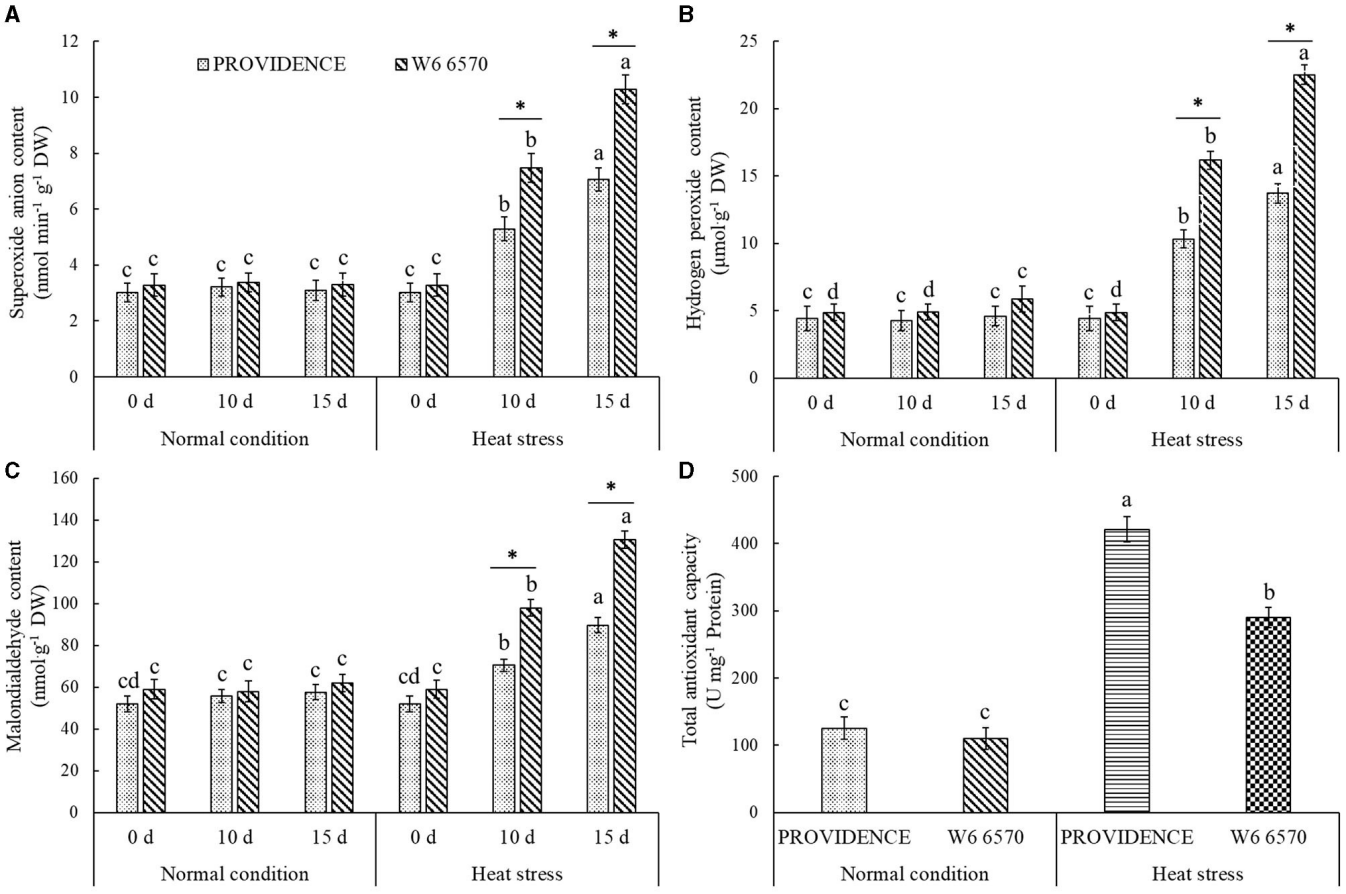
Changes in transcript levels of **(A)** superoxide anion content, **(B)** hydrogen peroxide content, **(C)** malondialdehyde content, and **(D)** total antioxidant capacity in the leaves of two creeping bentgrass materials (PROVIDENCE and W6 6570) under normal conditions or heat stress (38/33°C, day/night) in controlled growth chambers. Vertical bars indicate ± SE of mean (*n* = 4). Different letters above column indicate significant differences (*P* ≤ 0.05) in a particular material (PROVIDENCE or W6 6570) under normal conditions and heat stress. The asterisk “*” indicates significant differences (*P* ≤ 0.05) between PROVIDENCE and W6 6570 at one specific time point (3 h, 3 day, or 10 day).

**Figure 6:**
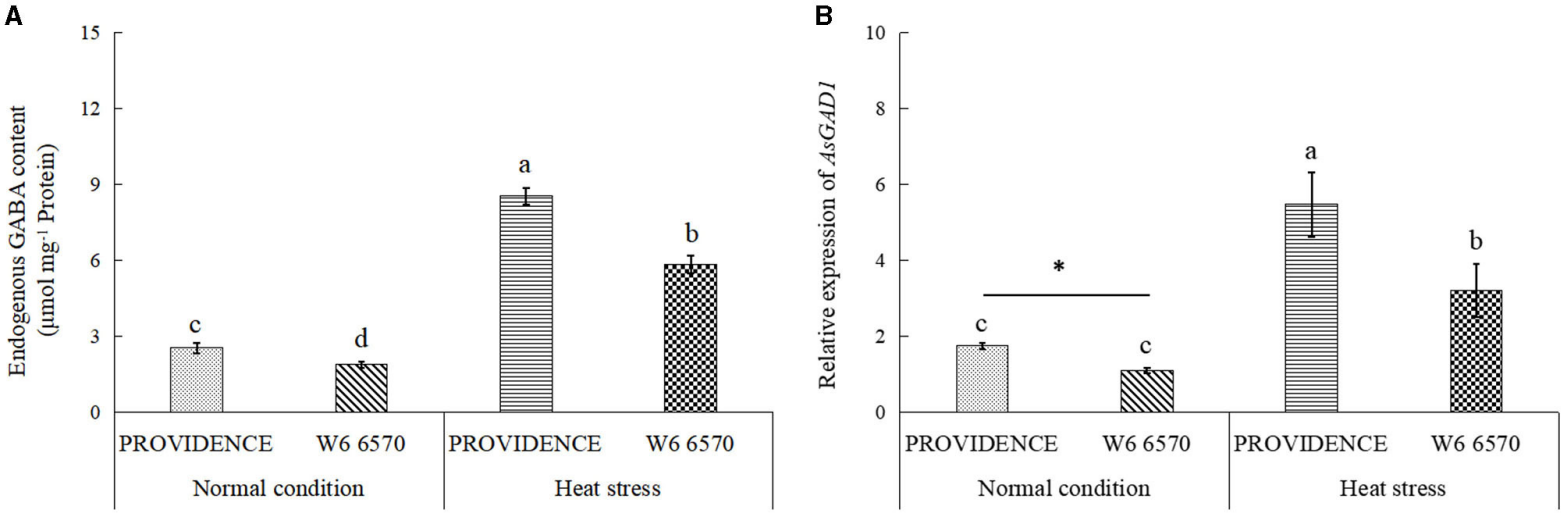
Changes in **(A)** endogenous γ-aminobutyric acid (GABA) content and **(B)** relative expression of *AsGAD1* gene in the leaves of two creeping bentgrass materials (PROVIDENCE and W6 6570) in response to 15 days of heat stress (38/33°C, day/night) in controlled growth chambers. Vertical bars indicate ± SE of mean (*n* = 4), and different letters above column indicate significant differences (*P* ≤ 0.05).

**Figure 7:**
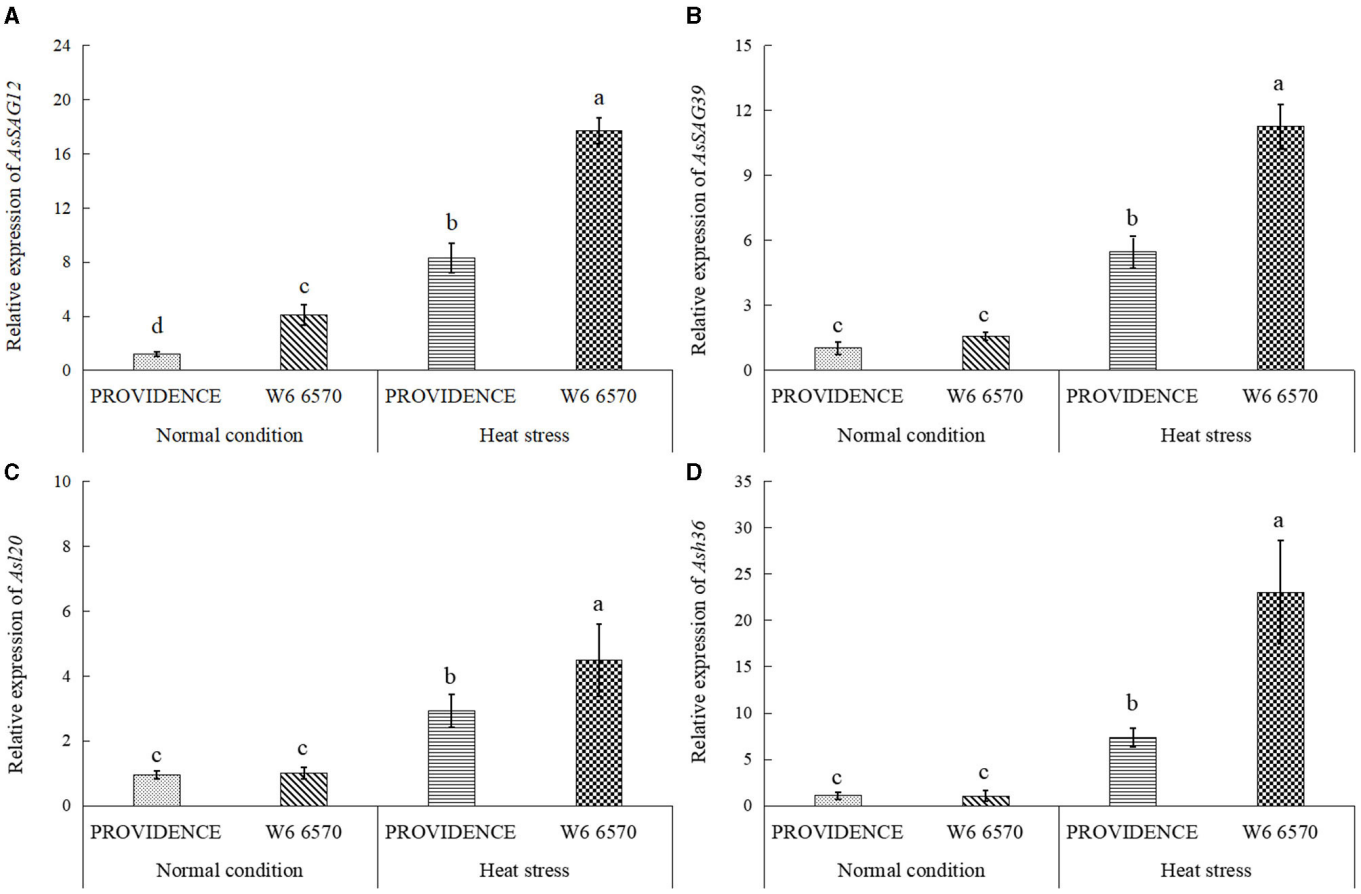
Changes in transcript levels of **(A)** *AsSAG12*, **(B)** *AsSAG39*, **(C)** *Asl20*, and **(D)** *Ash36* in the leaves of two creeping bentgrass materials (PROVIDENCE and W6 6570) in response to 15 days of heat stress (38/33°C, day/night) in controlled growth chambers. Vertical bars indicate ± SE of mean (*n* = 4), and different letters above column indicate significant differences (*P* ≤ 0.05).

There were no significant differences in O_2_^.−^, H_2_O_2_, and MDA content between PROVIDENCE and W6 6570 under normal growth conditions, but heat stress significantly increased their accumulations in both materials (Figures 5A–C). W6 6570 exhibited a 41 or 45% increase in O_2_^.−^ content compared with PROVIDENCE on the 10th or 15th day of heat stress, respectively (Figure 5A). Similarly, W6 6570 showed a significantly higher accumulation of H_2_O_2_ relative to PROVIDENCE on the 10th and 15th day of heat stress (Figure 5B). A 28 or 31% decrease in MDA content was found in PROVIDENCE as compared with that in W6 6570 on the 10th or 15th day of heat stress, respectively (Figure 5C). Heat stress induced a significant increase in TAC in PROVIDENCE and W6 6570, but the increase was more pronounced in PROVIDENCE. However, no significant difference in TAC existed between the two materials under normal conditions (Figure 5D). Endogenous GABA content and *AsGAD1*expression showed the same trend between PROVIDENCE and W6 6570 in response to heat stress, and significantly higher GABA content and *AsGAD1*expression were observed in PROVIDENCE than in W6 6570 under normal and heat conditions (Figures 6A,B). The expression of *AsSAG12* was significantly upregulated by heat stress, but PROVIDENCE had a significantly lower expression level of *AsSAG12* than W6 6570 under normal and heat conditions (Figure 7A). Expression levels of *AsSAG39, Asl20*, and *Ash36* were not different between PROVIDENCE and W6 6570 under normal conditions and upregulated by heat stress (Figures 7B,C). W6 6570 showed a 106, 54, or 211% increase in the expression level of *AsSAG39, Asl20*, or *Ash36* compared with PROVIDENCE under heat stress, respectively (Figures 7B–D).

## Discussion

Cool-season plant species are highly vulnerable to consistent high temperature stress. However, most cool-season grasses, such as perennial ryegrass, Kentucky bluegrass (*Poa pratensis*), and tall fescue, exhibit significant variations in tolerance to heat stress because of the genetic diversity of the population (Minner et al., 1983; Imada et al., 1993; Sun et al., 2014). Rapid and effective screening for heat-tolerant germplasms is of great value for breeding programs and also provides a guide for cultivation in warm regions. Cell membrane stability, as demonstrated by change in EL level, and Chl content have been widely used as critical and effective parameters to evaluate the variation in thermotolerance in different plant species (Sun et al., 2014; Rehman et al., 2016; Sharma et al., 2016). PI_ABS_ is considered a major indicator of leaf health status, because it synthetically reflects maximum photochemical efficiency and the number of activated photochemical reaction centers of photosystem II (PSII) in plant cells (Dai et al., 2019). In response to heat stress, the heat-tolerant maize (*Zea mays*) genotype “DKC7221” has the ability to maintain significantly higher PI_ABS_ than the heat-susceptible “P3167” (Doru, 2021). RWC and OP are the main parameters for evaluating leaf water status and osmoregulation ability. Terminal and persistent heat stress induces water deficit and imbalance in osmoregulation in leaves on account of acceleratory transpiration for heat emission and metabolic disturbance. Our previous study has proved that heat-induced water loss and decline in osmotic adjustment in leaves could be effectively alleviated by the exogenous application of GABA contributing to enhanced thermotolerance in creeping bentgrass (Li et al., 2016). Based on an integrated analysis of five physiological parameters (EL, Chl content, PI_ABS_, RWC, and OP), notable genetic variations were found among 41 creeping bentgrass materials, and LOFTS L-93, PROVEINDENCE, and 13M exhibited better heat tolerance, but PENNEAGLE, W6 6569, and W6 6570 had worse heat tolerance than other materials. Pearson correlation coefficients analysis showed that Chl content, OP, or EL had first, second, or third highest correlation coefficient with the SFVA ranking of heat tolerance among the 41 creeping bentgrass accessions, respectively. A recent study on natural variation of heat tolerance among 98 perennial ryegrass accessions also demonstrated that EL and Chl content were the top two evaluation parameters that exhibited high correlation coefficients with heat tolerance (Zhang et al., 2020). Thus, Chl and EL could be used as critical traits for a quick identification of heat tolerance among a large number of cool-season grass germplasms.

Greenhouse or growth chamber test under controlled environment conditions provides relatively stable heat stress in day and night, but high temperature under summer stress is fluctuant in nature and is accompanied by other complex factors such as changes in wind, air humidity, and solar ultraviolet radiation (Gaffen and Ross, 1998; Wang and Gaffen, 2001; Zhou et al., 2021). Therefore, crops that have stronger tolerance to fluctuant high temperature in summer are of great practical value. Eighteen creeping bentgrass materials with better heat tolerance selected from the controlled experiment in growth chambers were further tested under field conditions in the summer of 2020. The findings showed that significant variations in EL, Chl content, PI_ABS_, Fv/Fm, and TQ were found in the 18 materials under high temperature stress (daily maximum average temperature above 30°C for 32 days) in summer, in agreement with chamber-grown plants under controlled conditions. It has been reported that PI_ABS_ could act as an efficient breeding trait for the selection of heat-tolerant maize in field environments (Galic et al., 2019). In addition, TQ and Chl content could explain 40% genetic variations in the heat tolerance of 120 tall fescue accessions (Sun et al., 2014). In this study, 13M, PROVIDENCE, and LOFTS L-93 were identified as the most tolerant materials, and ProCup, MSCB-19, and NATIONAL had less heat tolerance than the other materials according to the SFVA using five parameters (EL, Chl content, PIABS, Fv/Fm, and TQ) in the field. Although the general raking trends between field test and the evaluation in growth chambers were similar, some differences were still not ignored. An earlier study suggested that laboratory screening test could be an accurate and rapid approach for selecting Kentucky bluegrass and perennial ryegrass differing in heat tolerance, since the results from field testing followed similar trends as the results under artificial conditions using growth chambers (Minner et al., 1983). Common bean (*Phaseolus vulgaris*) genotypes with superior heat tolerance could be successfully identified through conjoint estimations in the field and in the greenhouse (Porch, 2006). These findings indicate that integrative screening by laboratory and field tests could provide a more accurate evaluation of adaptation to high temperature in summer, because summer stress in the field depends on a particular location, weather, and soil condition.

A long-term period of heat stress retarded photosynthetic process and plant growth associated with the loss of Chl, which is the most important pigment for light harvest and transportation. Accompanied by accelerated leaf chlorosis under heat stress, significant declines in Pn and photochemical efficiency of PSII have been reported in creeping bentgrass and other plant species (Liu et al., 2019; Luo et al., 2020). Our current findings demonstrated that PROVIDENCE, having better heat tolerance, maintained significantly higher Chl, Pn, Fv/Fm, and PI_ABS_ than the heat-susceptible W6 6570 under optimal and high temperature conditions, indicating a significant genetic variation in Chl and photosynthesis between them. It has been reported that the maintenance of Chl cycle and photosynthetic complexes contributed to delayed leaf senescence in rice (*Oryza sativa*) (Krishna et al., 2016). For Chl anabolism, four prophobilinogen subunits are combined together into a tertapyrrole ring by PBGD; Mg-CHT is responsible for the insertion of Mg^2+^; POR catalyzes the formation of chlorophyllide (Masuda and Fujita, 2008). PPH is a key Chl-catabolic enzyme that catalyzes the removal of phytol chain from pheophytin (Schelbert et al., 2009). Heat stress attenuated Chl biosynthesis in the leaves of both PROVIDENCE and W6 6570, but PROVIDENCE showed better performance than W6 6570 through the maintenance of significantly higher expression levels of Chl-anabolic genes (*AsCHLH, AsPBGD*, and *AsPOR*) and lower Chl-catabolic gene *AsPPH* under heat stress. A previous study has confirmed that a delay in heat-induced leaf senescence could be modulated by gene expression involved in Chl biosynthesis (Luo et al., 2020). Suppression or decrease in *PPH* expression or encoding enzyme activity played a critical role in alleviating leaf senescence induced by heat stress in cool-season grass species (Jespersen et al., 2016; Jing et al., 2019; Rossi et al., 2020). Transcript levels of senescence-associated genes (*As120, Ash12, AsSAG12*, and *AsSAG39*) also indicated that PROVIDENCE exhibited delayed senescence as compared with W6 6570 after 15 days of heat stress.

Reactive oxygen species detoxification by an antioxidant system is one of the basic mechanisms for anti-aging in plants and animals (Munné-Bosch and Alegre, 2004; Pierpaola et al., 2016). The overaccumulation of detrimental ROS in stressed plants is accompanied by oxidative damage to proteins and membrane lipids, bringing about the disruption of chloroplast structure (Farooq et al., 2019). Melatonin suppressed heat- and dark-induced leaf senescence through enhancing activities of ROS-scavenging enzymes, thereby decreasing oxidative damage to cells (Zhang et al., 2016; Jing et al., 2019). In addition, the enhancement of antioxidant defense and ROS scavenging modulated by melatonin contributed to the delay of dehydration-induced leaf senescence in tobacco (*Nicotiana tabacum*) (Chen et al., 2021). Leaf senescence could be also effectively mitigated by the exogenous application of cytokinin or mannose involved in the improvement of antioxidant systems and ROS scavenging in grass species under salt or drought condition (Ma et al., 2016; Zhao et al., 2020). Persistent heat stress induced gradual increases in O_2_^.−^, H_2_O_2_, and MDA content, indicating heat-induced oxidative damage to PROVIDENCE and W6 6570. However, less oxidative damage and higher TAC were detected in PROVIDENCE relative to W6 6570 during heat stress. Therefore, a genetic variation in antioxidant capacity and ROS scavenging could play a vital part in stay-green trait in creeping bentgrass species.

As a non-protein amino acid, GABA is involved in nitrogen balance, energy metabolism, and metabolic shift with other metabolites (Fait et al., 2008; Lee et al., 2021). Enhanced GABA accumulation or metabolism could contribute to better adaptation to abiotic stresses, such as heat stress, in different plant species (Li et al., 2019; Tang et al., 2020; Rossi et al., 2021). The catabolism of GABA produces glutamic acid, which is an important biosynthetical precursor of Chl (Bouché and Fromm, 2004). An exogenous supply of GABA or chitosan improved endogenous GABA content, thereby alleviating salt- or drought-induced Chl loss in leaves of creeping bentgrass or white clover (Li et al., 2017, 2020). Despite the role of GABA as a metabolite for nutrition, metabolic homeostasis, and osmotic adjustment, increasing evidence indicates that the GABA plays a signaling molecule involved in stress response in plants (Fromm, 2020). GABA induced a significant improvement in antioxidant enzyme activities and upregulated stress-defensive genes encoding heat shock proteins, dehydrins, aquaporins, and osmotin against heat stress damage in sunflower (*Helianthus annuus*) (Abdel Razik et al., 2021). Khan et al. summarized the critical role of GABA in plant senescence *via* the regulation of oxidative damage, cytosolic pH, and carbon-nitrogen pool (Khan et al., 2021). A recent study also found that the suppression of *PPH* expression by GABA could significantly inhibit heat-caused Chl breakdown in creeping bentgrass (Rossi et al., 2020). In response to heat stress, endogenous GABA content and the expression of *AsGAD1* involved in GABA biosynthesis significantly increased in both the PROVIDENCE and W6 6570 genotypes. More importantly, PROVIDENCE maintained a significantly higher endogenous GABA content and the expression of *AsGAD1* under normal conditions and heat stress. Genetic variation in GABA could be beneficial in mitigating aging-induced leaf senescence and heat-induced detrimental effects, such as leaf chlorosis and metabolic disturbance, to achieve a stay-green phenotype. However, it still requires further investigations to uncover the role of GABA in leaf senescence based on genetic, metabolic, and molecular study.

## Conclusion

There were significant genetic variations in physiological traits (EL, RWC, OP, Chl content, Fv/Fm, PI_ABS_, and TQ) among 41 creeping bentgrass accessions that originated from 15 different countries. The 13M, the PROVIDENCE, and the LOFTS L-93 were the top three cultivars with better tolerance to high temperature and summer stress than other materials based on laboratory and field tests. In response to heat stress, stay-green genotype PROVIDENCE exhibited significantly higher photochemical efficiency, Pn, Tr, and WUE than W6 6570 with susceptibility to heat damage. Delayed leaf senescence associated with less Chl loss was detected in PROVIDENCE *via* the maintenance of significantly higher expression levels of Chl-anabolic genes (*AsCHLH, AsPBGD*, and *AsPOR*) and lower expression level of Chl-catabolic gene *AsPPH* under heat stress. Genetic attributes of better ROS-scavenging capacity and higher endogenous GABA content could play positive roles in alleviating heat-induced senescence, oxidative damage, and metabolic disturbance in PROVIDENCE. Genetic and molecular mechanisms are still required to elucidate the relationship between stay-green variation and heat tolerance in creeping bentgrass species.

## Data Availability Statement

The original contributions presented in the study are included in the article/supplementary material, further inquiries can be directed to the corresponding author/s.

## Author Contributions

ZL and YP: conceived and designed the research. MT: conducted the experiments. MT and ZL: evaluated the data. LH, ZL, and YP: provided different chemical reagents and experimental material. Article writing was completed by ZL. MT, LH, YP, YZ, and MH: reviewed and edited the manuscript. All authors contributed to the article and approved the submitted version.

## Funding

This research was supported by China Postdoctoral Science Foundation (2021T140058) and the Sichuan Forage Innovation Team Project of the Industrial System Construction of Modern Agriculture of China (sccxtd-2020-16).

## Conflict of Interest

The authors declare that the research was conducted in the absence of any commercial or financial relationships that could be construed as a potential conflict of interest.

## Publisher's Note

All claims expressed in this article are solely those of the authors and do not necessarily represent those of their affiliated organizations, or those of the publisher, the editors and the reviewers. Any product that may be evaluated in this article, or claim that may be made by its manufacturer, is not guaranteed or endorsed by the publisher.
