# Supplementary material for: Adaptability to High Temperature and Stay-Green Genotypes Associated With Variations in Antioxidant, Chlorophyll Metabolism, and γ-Aminobutyric Acid Accumulation in Creeping Bentgrass Species
Source: Front Plant Sci. 2021 Oct 28;12:750728. doi: 10.3389/fpls.2021.750728 (PMC8581182; doi:10.3389/fpls.2021.750728)
Supplement: Supplementary file 1 [file Data_Sheet_1.docx]

**
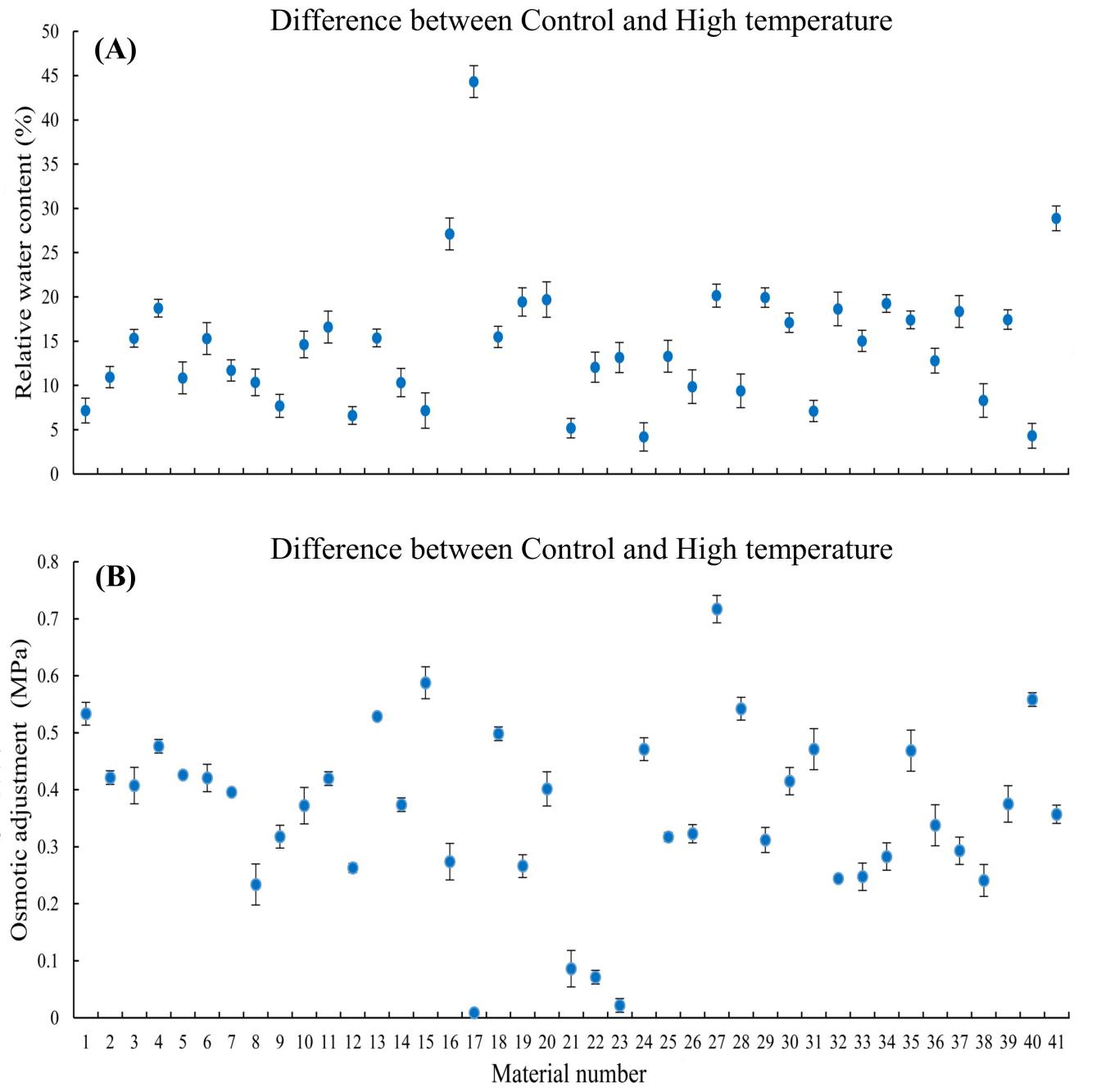
**

**Fig. S1** The difference in (A) relative water content or (B) osmotic potential between control and high temperature of 41 creeping bentgrass materials. Vertical bars indicate ± SE of mean (n=4).


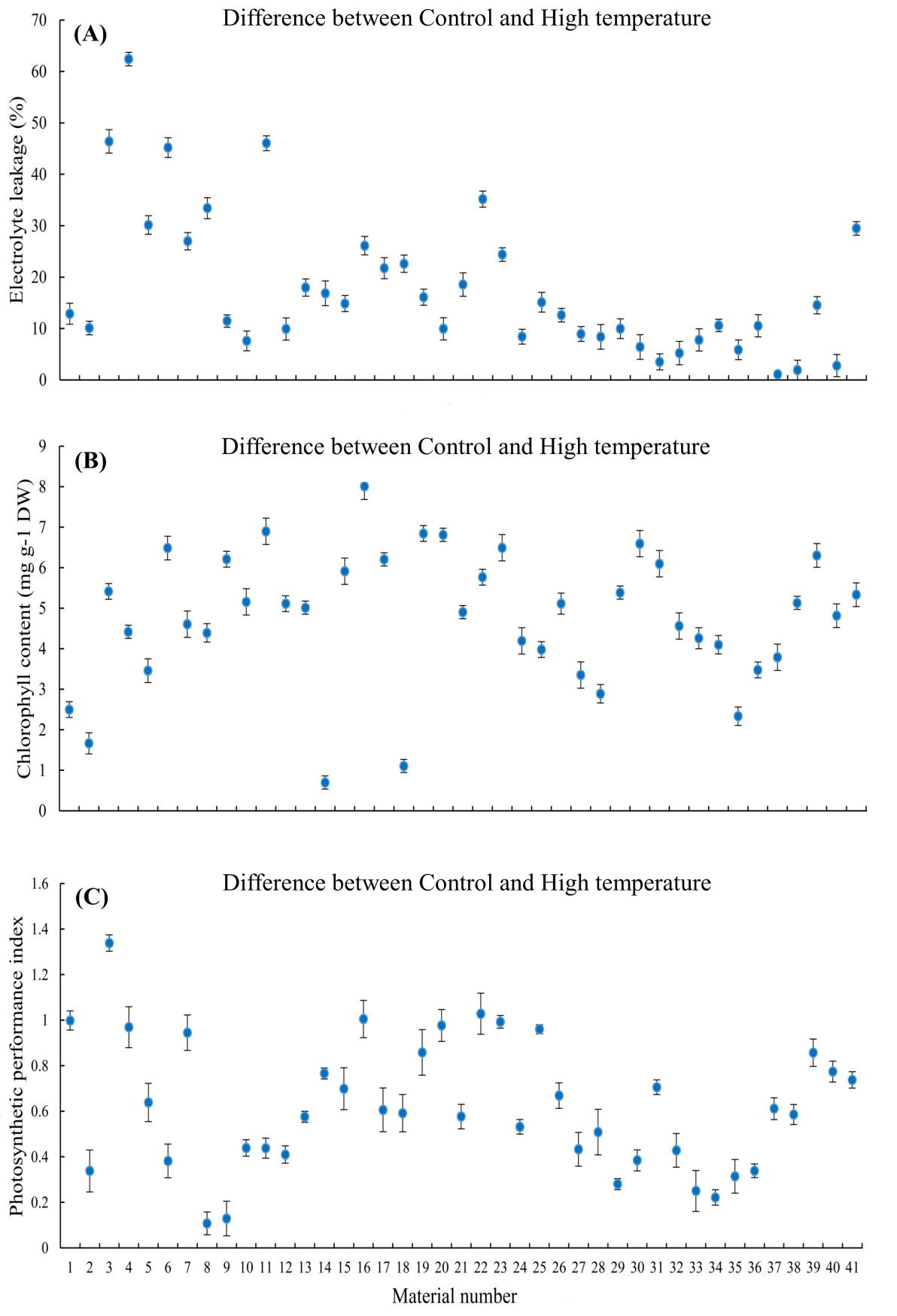


**Fig. S2** The difference in (A) electrolyte leakage, (B) chlorophyll content, or (C) photosynthetic performance index between control and high temperature of 41 creeping bentgrass materials. Vertical bars indicate ± SE of mean (n=4).


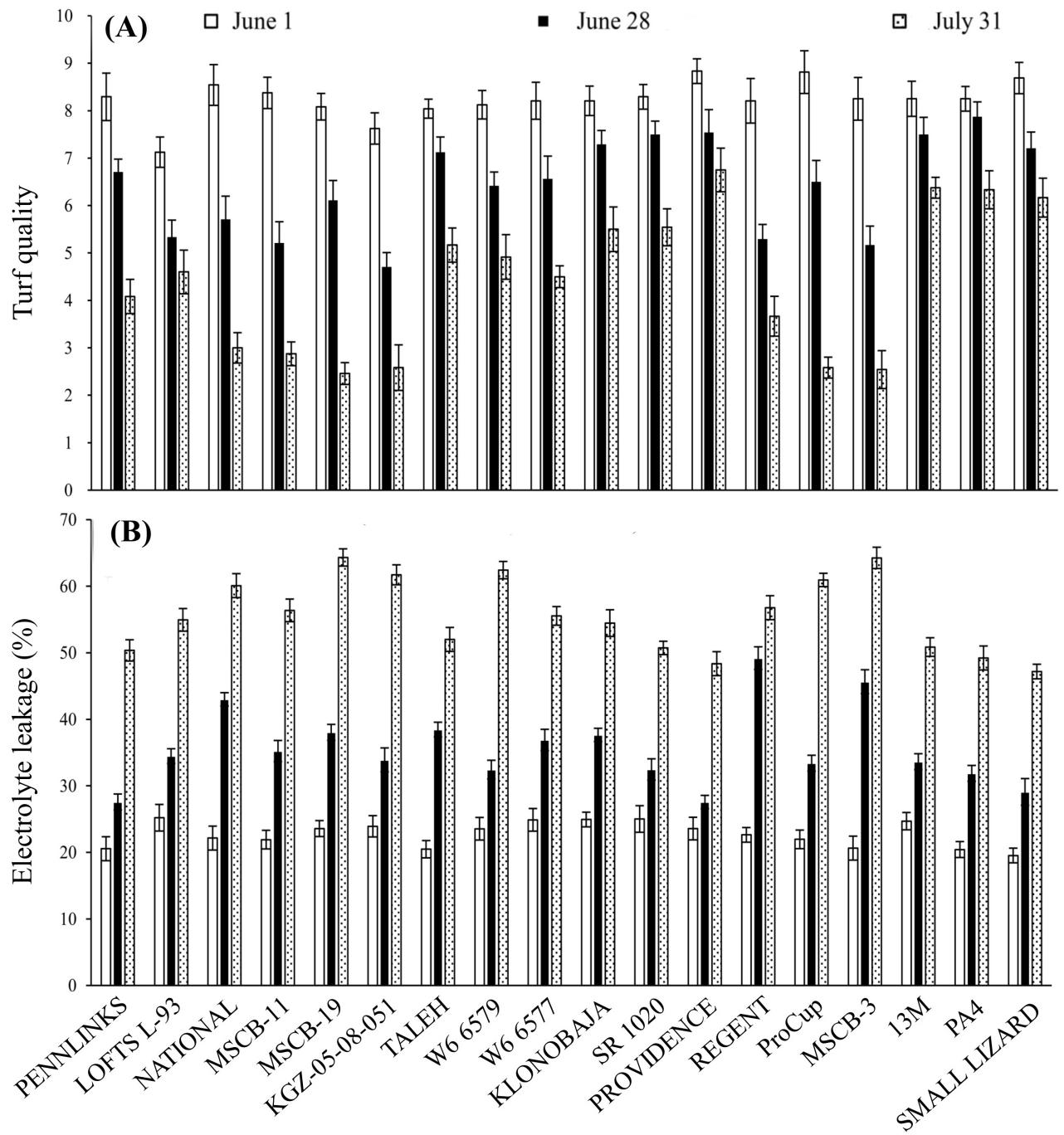


**Fig. S3** Changes in (A) turf quality and (B) electrolyte leakage of 18 creeping bentgrass materials during summer in 2020 under field condition. Vertical bars indicate ± SE of mean (n=4). Maximum, minimum, and average air temperature were 24.5°C, 19.2°C, and 22.8°C on June 1, 30.1°C, 22.9°C, and 25.7°C on June 28, or 24.8°C, 22.2°C, and 23.1°C on July 31 , respectively.


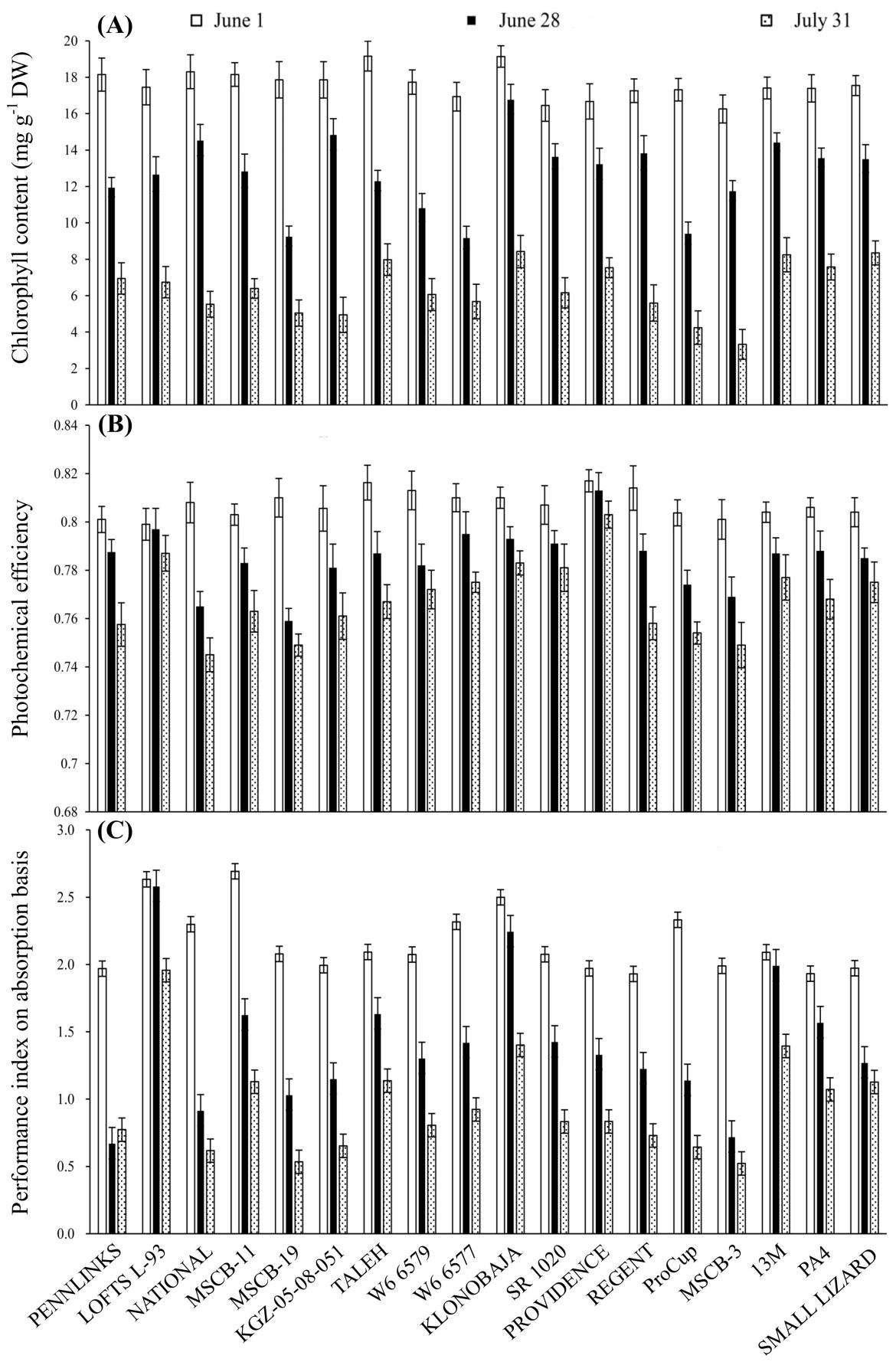


**Fig. S4** Changes in (A) chlorophyll content, (B) photochemical efficiency, and (C) performance index on absorption basis of 18 creeping bentgrass materials during summer in 2020 under field condition. Vertical bars indicate ± SE of mean (n=4). Maximum, minimum, and average air temperature were 24.5°C, 19.2°C, and 22.8°C on June 1, 30.1°C, 22.9°C, and 25.7°C on June 28, or 24.8°C, 22.2°C, and 23.1°C on July 31 , respectively.
