# Supplementary material for: Adaptability to High Temperature and Stay-Green Genotypes Associated With Variations in Antioxidant, Chlorophyll Metabolism, and γ-Aminobutyric Acid Accumulation in Creeping Bentgrass Species
Source: Front Plant Sci. 2021 Oct 28;12:750728. doi: 10.3389/fpls.2021.750728 (PMC8581182; doi:10.3389/fpls.2021.750728)
Supplement: Supplementary file 2 [file Data_Sheet_2.docx]

**Table S1** Cultivar names or accession numbers provided by NPGS and origin areas of 42 creeping bentgrass materials.

| Material number | Cultivar name or Accession number | Origin |  | Material number | Cultivar name or Accession number | Origin |
| --- | --- | --- | --- | --- | --- | --- |
| 1 | PENNLINKS | PA, USA |  | 22 | W6 6574 | Uzbekistan |
| 2 | LOFTS L-93 | USA |  | 23 | O93 | Spain |
| 3 | PENNEAGLE | PA, USA |  | 24 | KLONOBAJA | Ukraine |
| 4 | MSCB-10 | MS, USA |  | 25 | CARMEN | Holland |
| 5 | BLUETAG SEASIDE | OH, USA |  | 26 | KESZTHELYI 4 | Hungary |
| 6 | EMERALD | MD, USA |  | 27 | SR 1020 | OR, USA |
| 7 | UKR-99-179 | Ukraine |  | 28 | PROVIDENCE | OR, USA |
| 8 | NATIONAL | OR, USA |  | 29 | PUTTER | ID, USA |
| 9 | MSCB-11 | MS, USA |  | 30 | SOUTHSHORE | Holland |
| 10 | MSCB-19 | MS, USA |  | 31 | REGENT | USA |
| 11 | PENNCROSS | USA |  | 32 | SEASIDE | OR, USA |
| 12 | KGZ-05-08-051 | Kyrgyzstan |  | 33 | ProCup | USA |
| 13 | W6 6575 | Turkey |  | 34 | MSCB-3 | MS, USA |
| 14 | TALEH | Afghanistan |  | 35 | 13M | USA |
| 15 | W6 6579 | Austria |  | 36 | PA4 | USA |
| 16 | W6 6569 | Afghanistan |  | 37 | PENN WAY | USA |
| 17 | W6 6570 | Iran |  | 38 | PA1 | USA |
| 18 | W6 6577 | Switzerland |  | 39 | KROMI | Denmark |
| 19 | W6 6578 | Sweden |  | 40 | SMALL LIZARD | USA |
| 20 | W6 6571 | Germany |  | 41 | SEASIDE 2 | USA |
| 21 | F-251 | Spain |  |  |  |  |

**Table S2** Primer sequences and relative information of analyzed genes in creeping bentgrass.

| Target Gene | Forward Primer (5’-3’) | Reverse Primer (5’-3’) | Tm (◦C) |
| --- | --- | --- | --- |
| *Asβ-actin* | CCTTTTCCAGCCATCTTTCA | GAGGTCCTTCCTGATATCCA | 58 |
| *AsPBGD* | TAGCGCTGCGGATTAGAACT | GAAGGATAACGAACCGCTGA | 55 |
| *AsCHLH* | CATCAGGGCGGATAGAGAGA | TCTGCCACAATCAGCTTCAG | 56 |
| *AsPPH* | GAATGTCATTGCCGTCTGAA | CAATGAAATGCTGGACCTGA | 55 |
| *AsPOR* | GCGTCTACTGGAGCTGGAAC | GTCACTTCATGCAGGTCACG | 58 |
| *AsGAD1* | CCGAGTGCGACARGCTCATC | CGGCYGAASTCYTCCCKGA | 58 |
| *Asl20* | GGGTAGACGGCAACGATACT | TACTTGGTTGAATCGTCGGA | 58 |
| *Ash36* | TGGGAATGTGTTCAGGGTAA | TCACCTCGATGAGGTAGTCG | 58 |
| *AsSAG12* | GTGAAGAACCAGGGCCAGTG | ACAGTCCACCAACTCCTGCT | 60 |
| *AsSAG39* | CCTCGCTGTTCTTGCCGTGAG | CGTGCTCAGCCATCCACTTCTC | 60 |
